# A different perspective on studying stroke predictors: joint models for longitudinal and time-to-event data in a type 2 diabetes mellitus cohort

**DOI:** 10.1186/s12933-025-02713-9

**Published:** 2025-04-16

**Authors:** F. J. San Andrés-Rebollo, J. Cárdenas-Valladolid, J. C. Abanades-Herranz, P. Vich-Pérez, J. M. de Miguel-Yanes, M. Guillán, M. A. Salinero-Fort, the MADIABETES, A. M. Sobrado-de Vicente-Tutor, M. Sanz-Pascual, M. Arnalte-Barrera, S. Pulido-Fernández, E. M. Donaire-Jiménez, C. Montero-Lizana, M. Domínguez-Paniagua, P. Serrano-Simarro, R. Echegoyen-de Nicolás, P. Gil-Díaz, I. Cerrada-Somolinos, R. Martín-Cano, A. Cava-Rosado, T. Mesonero-Grandes, E. Gómez-Navarro, A. Maestro-Martín, A. Muñoz-Cildoz, M. E. Calonge-García, M. Martín-Bun, P. Carreño-Freire, J. Fernández-García, A. Morán-Escudero, J. Martínez-Irazusta, E. Calvo-García, A. M. Alayeto-Sánchez, C. Reyes-Madridejos, M. J. Bedoya-Frutos, B. López-Sabater, J. Innerarity-Martínez, A. Rosillo-González, A. I. Menéndez-Fernández, F. Mata-Benjumea, C. Martín-Madrazo, M. J. Gomara-Martínez, C. Bello-González, A. Pinilla-Carrasco, M. Camarero-Shelly, A. Cano-Espin, J. Castro Martin, B. de Llama-Arauz, A. de Miguel-Ballano, M. A. García-Alonso, J. N. García-Pascual, M. I. González-García, C. López-Rodríguez, M. Miguel-Garzón, M. C. Montero-García, S. Muñoz-Quiros-Aliaga, S. Núñez-Palomo, O. Olmos-Carrasco, N. Pertierra-Galindo, G. Reviriego-Jaén, P. Rius-Fortea, G. Rodríguez-Castro, J. M. San Vicente-Rodríguez, M. E. Serrano-Serrano, M. M. Zamora-Gómez, M. P. Zazo-Lázaro

**Affiliations:** 1 Las Calesas Health Centre, Madrid, Spain; 2 Biosanitary Research and Innovation Foundation of Primary Care (FIIBAP), Madrid, Spain; 3 Frailty, Multimorbidity Patterns and Mortality in the Elderly Population Residing in the Community– Hospital La Paz Institute for Health Research IdiPAZ, Madrid, Spain; 4 Alfonso X El Sabio University, Madrid, Spain; 5 Monóvar Health Centre, Madrid, Spain; 6 Los Alpes Health Centre, Madrid, Spain; 7 Internal Medicine Department, Hospital General Universitario Gregorio Marañón, Instituto de Investigación Sanitaria Gregorio Marañón (IiSGM), Universidad Complutense de Madrid, Madrid, Spain; 8 Department of Neurology, Neurovascular Unit, Fundación Jiménez Díaz University Hospital, Madrid, Spain; 9 Network for Research on Chronicity, Primary Care, and Health Promotion (RICAPPS), Madrid, Spain

**Keywords:** Stroke, Risk factors, Follow-up studies, Joint models, Type 2 diabetes mellitus, Prediction models

## Abstract

**Background:**

Most predictive models rely on risk factors and clinical outcomes assessed simultaneously. This approach does not adequately reflect the progression of health conditions. By employing joint models of longitudinal and survival data, we can dynamically adjust prognosis predictions for individual patients. Our objective was to optimize the prediction of stroke or transient ischemic attack (TIA) via joint models that incorporate all available changes in the predictive variables.

**Methods:**

A total of 3442 patients with type 2 diabetes mellitus (T2DM) and no history of stroke, TIA or myocardial infarction were followed for 12 years. Models were constructed independently for men and women. We used proportional hazards regression models to assess the effects of baseline characteristics (excluding longitudinal data) on the risk of stroke/TIA and linear mixed effects models to assess the effects of baseline characteristics on longitudinal data development over time. Both submodels were then combined into a joint model. To optimize the analysis, a univariate analysis was first performed for each longitudinal predictor to select the functional form that gave the best fit via the deviance information criterion. The variables were then entered into a multivariate model using pragmatic criteria, and if they improved the discriminatory ability of the model, the area under the curve (AUC) was used.

**Results:**

During the follow-up period, 303 patients (8.8%) experienced their first stroke/TIA. Age was identified as an independent predictor among males. Among females, age was positively associated with atrial fibrillation (AF). The final model for males included AF, systolic blood pressure (SBP), and diastolic blood pressure (DBP), with albuminuria and the glomerular filtration rate (GFR) as adjustment variables. For females, the model included AF, blood pressure (BP), and renal function (albuminuria and GFR), with HbA1c and LDL cholesterol as adjustment variables. Both models demonstrated an AUC greater than 0.70.

**Conclusions:**

Age, AF, and SBP have been confirmed as significant predictive factors in both sexes, whereas renal function was significant only in women. Interestingly, an increase in DBP may serve as a protective factor in our cohort. These factors were particularly relevant in the last 3–7 years of follow-up.

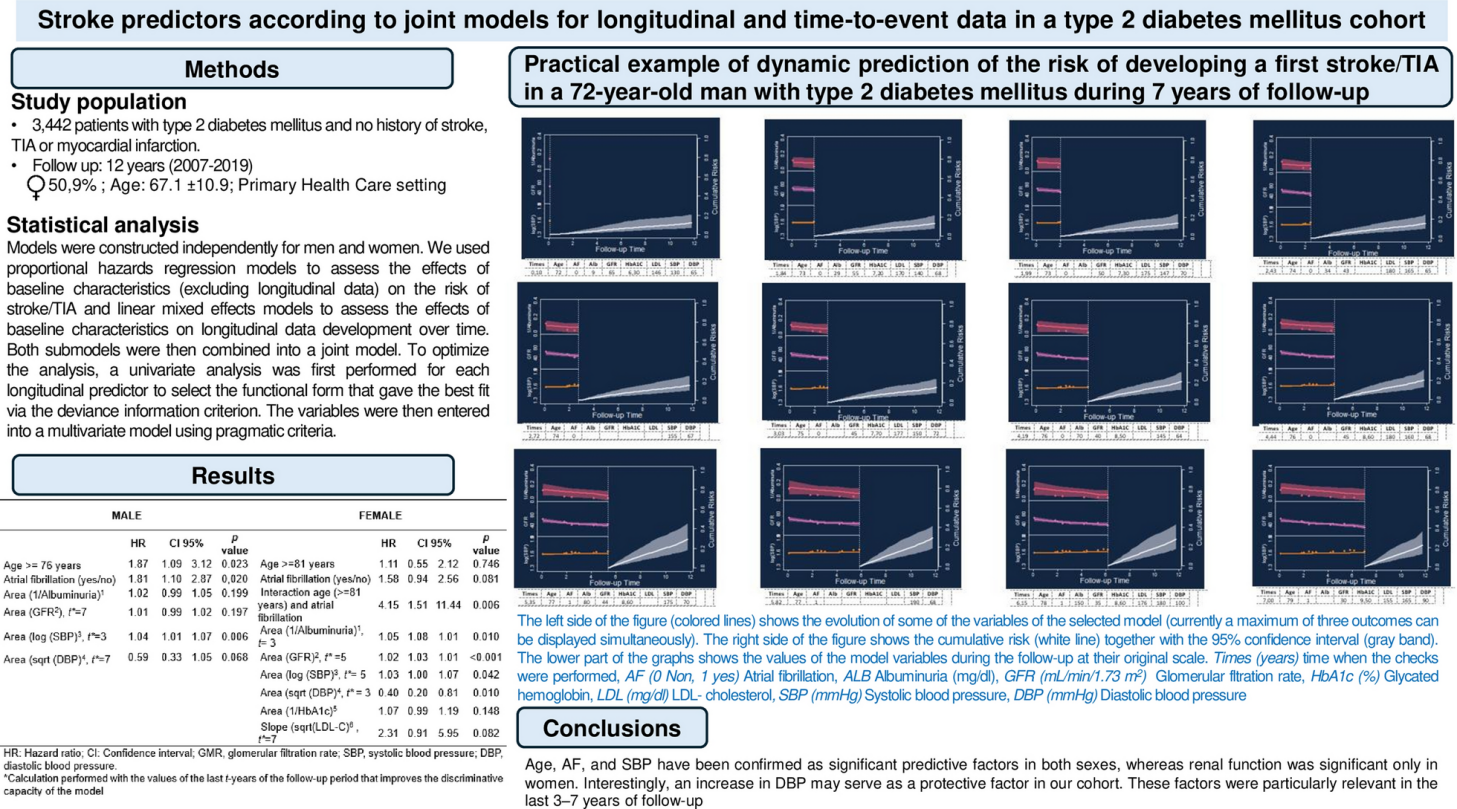

**Supplementary Information:**

The online version contains supplementary material available at 10.1186/s12933-025-02713-9.

## Background

Stroke is a major public health problem worldwide. It is the second most common cause of death [1] and the third most common cause of disability-adjusted life years [2]. Between 1990 and 2019, the number of deaths attributable to ischemic stroke worldwide increased from 2.04 million to 3.29 million, and this figure is expected to increase to 4.90 million by 2030 [3].

The prevalence of stroke in Europe is 9.2%, and the incidence adjusted for sex is 191.9 per 100,000 person-years (95% confidence interval (CI) 156.4–227.3) according to a recent systematic review [4]. In Spain, 120,000 new strokes occur each year, making stroke the leading cause of disability among Spanish adults [5]. In the study by Díaz-Guzmán J et al., the incidence rates per 100,000 individuals standardized to the 2006 European population were as follows: all cerebrovascular events, 176 (95% CI 169–182); all stroke events (nontransitory ischemic attack), 147 (95% CI 140–153); transitory ischemic attack (TIA), 29 (95% CI 26–32); ischemic stroke, 118 (95% CI 112–123); and intracerebral hemorrhage, 23 (95% CI 21–26) [6]. The incidence rates apparently increased with age in both sexes, with a peak at or above 85 years of age.

Stroke is currently the second leading cause of death in Spain, after ischaemic heart disease, and the leading cause of death among women. According to data from the National Institute of Statistics (INE), cerebrovascular diseases caused 23,173 deaths in 2022 (47.9 per 100,000 individuals) (https://www.ine.es/jaxi/Datos.htm?tpx=67875).

The global prevalence of type 2 diabetes mellitus (T2DM) is 10.5% among adults aged 20–79 years according to the IDF Diabetes Atlas 2021 (10th edition) [7], and macrovascular complications such as stroke are more common among people with T2DM than among people with normal glucose metabolism [8].

Few reports have focused on the effects of traditional vascular risk factors such as hypertension, hypercholesterolemia, atrial fibrillation (AF), smoking, obesity and a sedentary lifestyle in people with T2DM, especially in large representative outpatient cohorts from southern Europe [9]. Furthermore, these studies use only a small fraction of the available information on risk factors. Although risk factors such as systolic blood pressure (SBP), lipids, and glycated hemoglobin (HbA1c) are measured repeatedly over time, risk scores are typically based on either baseline or the most recent available risk factor measurements. This approach does not account for changes in risk factors over time for each participant beyond differences between participants. Using the information obtained by repeated risk factor measurements could provide a better understanding of disease progression and improve risk prediction for the event of interest compared with a single punctual measurement.

We aimed to describe the factors associated with the risk of stroke in a well-characterized cohort of patients with T2DM (MADIABETES cohort) to determine whether different characteristics of longitudinal profiles are associated with the risk of cerebrovascular events via joint model analyses.

## Methods

The MADIABETES Study is a large, ongoing research project launched in 2007 to better understand the long-term impact of prevalent T2DM. The study initially enrolled 3,442 outpatients with T2DM in 2007, plus an additional 727 outpatients by the end of 2010. The participants were randomly selected from primary care centres in the metropolitan area of Madrid, Spain.

The current analysis includes data from 2008 to 2019. General practitioners carefully collected information at the start of the study and continued to do so annually during routine clinical visits. The data, which include baseline information and ongoing diagnoses, were collected from electronic medical records and hospital discharge reports.

The MADIABETES study has provided valuable insights into several variables, such as retinopathy, chronic kidney disease (CKD), body mass index (BMI), smoking, AF, and lipid values, and their associations with mortality, myocardial infarction, and stroke [9–14]. Published data from the MADIABETES study are consistent with findings from other national and international studies [15].

The inclusion criterion for the study was individuals aged 30 years and older with a previous diagnosis of T2DM. Patients with T1DM and those who were homebound were excluded.

T2DM was defined on the basis of a reported history of diabetes, use of diabetes medications, fasting plasma glucose levels, HbA1c, or results from an oral glucose tolerance test. Additionally, BMI, blood pressure, neuropathy, retinopathy, and smoking habits were assessed.

Stroke was defined as the rapid onset of clinical signs of focal or global loss of cerebral function that lasted > 24 h and/or could not be explained by other medical conditions and/or confirmed by brain computed tomography (CT) or magnetic resonance imaging (MRI), whereas TIA was defined as the occurrence of neurological symptoms or signs lasting < 24 h due to an episode of neurological dysfunction caused by focal cerebral, spinal cord, or retinal ischaemia without acute infarction. The stroke diagnosis included both hemorrhagic and ischemic stroke (covering all etiologic subtypes of ischemic stroke).

The vital status of each patient (dead or alive) was ascertained on December 31st, 2019, with data from the mortality records of the Spanish National Institute of Statistics (Instituto Nacional de Estadística, http://www.ine.es). Therefore, data concerning vital status and date and cause of death were available for all patients, with no loss of data during follow-up. The underlying cause of death stated on the death certificates was coded according to the International Statistical Classification of Diseases, Tenth Revision [16].

Hypertension was defined according to the 2017 ACC/AHA blood pressure guidelines as systolic blood pressure ≥ 140 mm Hg or diastolic blood pressure ≥ 90 mm Hg. [17]. Persistent atrial fibrillation was defined as the persistence of the rhythm disturbance, as assessed by history and ECG, on the basis of irregular RR intervals (when atrioventricular conduction is not impaired), the replacement of the steady P wave by rapid oscillations of fibrillatory waves that vary in size, shape and timing.

### Statistical analysis

Descriptive data are presented as the means and standard deviations, medians and interquartile ranges, percentiles, or box plots. Continuous variables were compared between two groups via the t test for normally distributed data and the Mann‒Whitney test for nonnormally distributed data. Categorical variables were compared via the chi-square test. We calculated the cumulative incidence of first stroke/TIA using the number of new cases as the numerator and the total initial population at risk as the denominator. We determined the number of patient-years at risk of developing a first stroke/TIA between the date of the baseline visit and the date of death, the date of the first stroke/TIA, or the end of the study period. We estimated incidence density as the number of new cases divided by patient-years at risk.

Conceptually, the joint model links a survival model with a suitable model for the endogenous longitudinal covariates [18, 19]. To assess changes in the levels of HbA1c, albuminuria, the glomerular filtrate rate (GFR), low-density cholesterol (LDL-C), systolic blood pressure (SBP) and diastolic blood pressure (DBP) over time (hereafter referred to as longitudinal predictors) while accounting for the correlation between the repeated measurements of each patient, we utilized the framework of linear mixed-effects models.

**Fig. 1:**
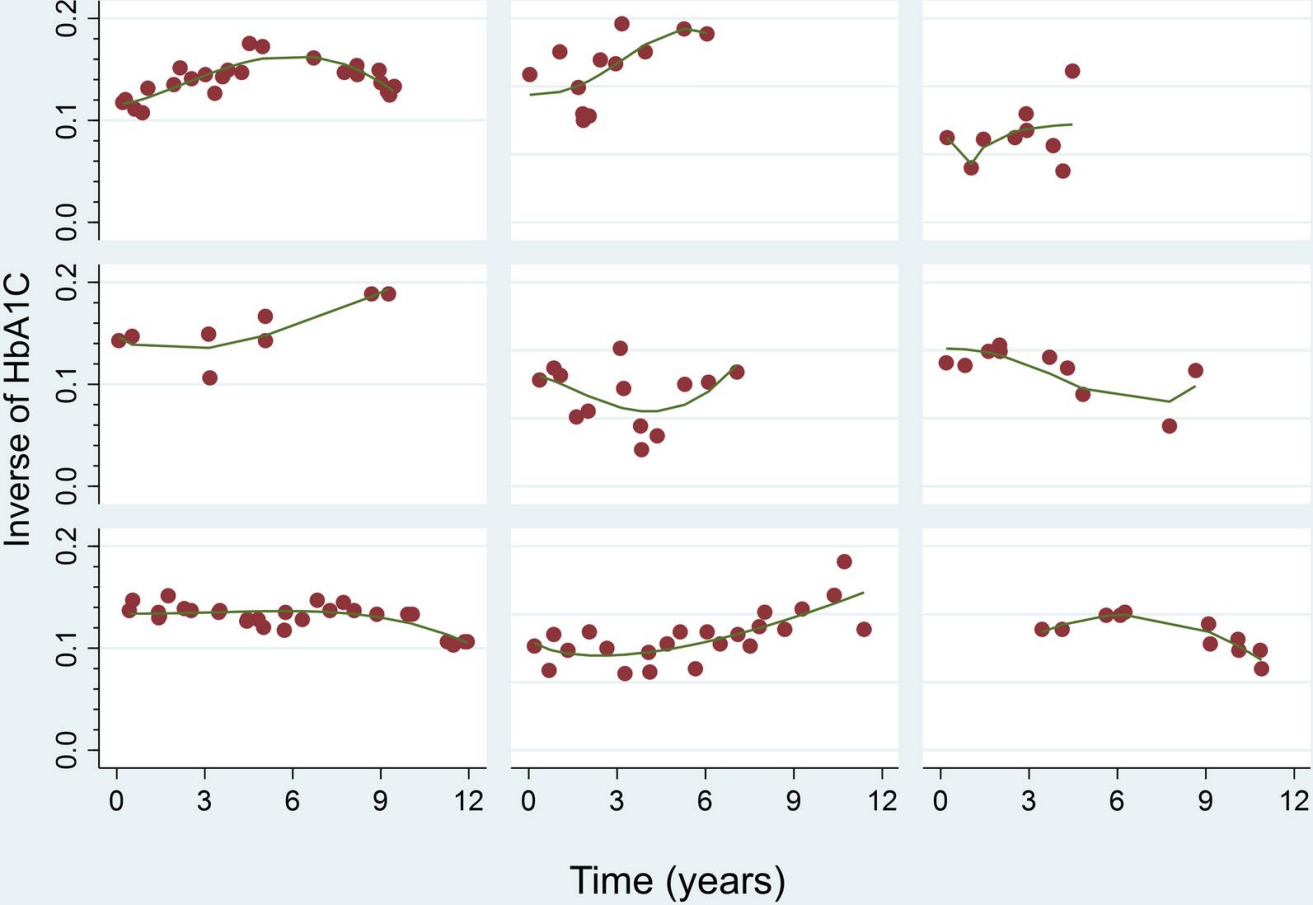
Longitudinal profiles of inverse (HbA1C) over time for nine randomly selected subjects from the data set

Given the nonlinearity in the longitudinal profile of all biomarkers (Fig. 1), we assumed a flexible linear mixed-effects submodel including natural cubic splines for time with two internal knots at 3 and 7 years (corresponding to 33.3% and 66.7% of the observed follow-up time; i.e., quantiles) in both the fixed-effects and random-effects parts of the longitudinal models, except for albuminuria, for which we used linear and quadratic terms. We controlled for baseline age, BMI, and time of onset of T2DM (using natural cubic splines with 3 degrees of freedom each), as well as the presence of AF, retinopathy, neuropathy, and peripheral arteriopathy. The appropriate random-effects structure that best fit the data were selected on the basis of likelihood ratio tests. We performed the omnibus test to assess the interaction terms of age with the other variables; if it was significant, then we looked for the terms that were significant. Finally, the variables with a significance level of *p* > 0.2 were eliminated from the models. All longitudinal variables were transformed (Supplementary Fig. 1) to normalize their distribution and to satisfy the principle of homoscedasticity.

Proportional hazards models were used for survival submodels. The baseline risk function was approximated via B-splines, with five knots assumed at the percentiles of the observed event times. The baseline predictors of the event of interest (i.e., stroke/TIA) tested in the models were age; duration of T2DM; BMI (< 25, 25–29.9, 30–35, > 35 kg/m^2^); and the presence of AF, retinopathy, neuropathy, and peripheral arteriopathy. The effect of age on stroke risk was nonlinear and was therefore categorized, with cut-off points of 81 years in women and 76 years in men providing the best fit and discriminatory ability to the model.

### Building and fitting a joint model by sex

The two classes of submodels described above are combined (joint modelling methodology). A critical point of this joint modelling is the high computational effort given the large volume of data involved [20, 21]. Therefore, to optimize the analysis, the model construction procedure followed pragmatic criteria.

Since we were interested in predicting risks rather than simply assessing the degree of association between the trend of repeated outcomes and the time-to-event, we went beyond the standard joint model, which uses only the latent value of the biomarkers or risk factors, and investigated whether the risk of an event could also be affected by the slope or a summary of the entire history of longitudinal outcomes (area) [22]. We first performed a univariate analysis for each of the longitudinal predictors to select the functional form that gave the best fit via the deviance information criterion (DIC).

We subsequently constructed a multivariate model using longitudinal predictors that showed a statistical significance level of *p* < 0.35 in the univariate analysis, which has been used in other studies, or risk factors widely accepted in the literature [23–25]. We subsequently assessed whether the discriminatory capacity of this first multivariate model improved with the inclusion of the variables not previously selected, as well as the interaction of age with the linear predictors. Finally, we searched for the most parsimonious model by removing the variables that, in addition to having a *p* > 0.2, did not represent an essential modification of the model fit and capacity. The convergence of the models was monitored via trace plots.

We evaluated the discriminative ability of all the models via the receiver operating characteristic (ROC) method. In all cases, the best area under the curve (AUC) was obtained by using information up to year seven of follow-up and predicting events occurring in a three-year window.

In addition to the AUC, the predictive accuracy of the final model was analysed via prediction error (PE) and calibration measures. Overfitting-corrected estimates of these measures were obtained via an internal validation procedure following other authors [26]. Optimism corrections were made via a bootstrapping method. The procedure described was the same for the two models by sex.

Assuming that most of the losses in the longitudinal outcome are noninformative (missing at random), there was no need to impute missing data in the context of joint models (which belong to the class of shared parameter models) [19].

The JMbayes2 (v.4.1) package of the R software (GNU General Public License, version 4.2.3) (www. cran.r- project. org) [27] was used to fit the joint models and to evaluate different association structures. SPSS (version 26.0; IBM Corp, Armonk, NY, USA) and STATA (version 16/SE; StataCorp, TC, USA) were used for the remaining analyses.

## Results

The study included 3442 people with T2DM who had never had a stroke, a TIA or a myocardial infarction. A total of 50.9% of the participants were women; the mean age was 67.1 years (standard deviation (SD): 10.9). Table 1 shows the main characteristics at baseline. There were significant differences between men and women with respect to age, duration of diabetes, AF, peripheral arteriopathy, and diabetic neuropathy. During the 29,994 person-years of follow-up, 303 cases of stroke or TIA occurred, resulting in an incidence rate of 1010 per 100,000 person-years of follow-up (95% CI 899–1131). There were no significant sex differences in the incidence rates of stroke or TIA, which were 1017 per 100,000 person-years (95% CI 859–1196) in men and 1004 per 100,000 person-years (95% CI 852–1174) in women.

**Table 1:** Baseline characteristics of the variables analysed in the models stratified by sex

	Females	Males	*p* value
	(n = 1754)	(n = 1688)	
Age (years), mean (SD)	68.9 (10.7)	65.3 (11.1)	< 0.001
[95% CI]	[68.4–69.4]	[64.8–65.8]	
Diabetes duration (years), median [IQR]	6.4 (4.6)	6.0 (4.6)	< 0.001
	[6.2–6.8]	[5.9–6.3]	
BMI (kg/m^2^), mean (SD) [95% CI]	31.1 (5.5)	29.7 (4.4)	< 0.001
	[30.9–31.4]	[29.5–29.9]	
< 25 kg/m^2^, % [95% CI]	11.3 [9.9–12.9]	12.4 [10.9–14.1]	< 0.001
25–30 kg/m^2^, % [95% CI]	34.1 [31.9–36.3]	45.6 [43.3–47.9]	
31–35 kg/m^2^, % [95% CI]	33.4 [31.2–35.6]	30.9 [28.8–33.2]	
> 35 kg/m^2^, % [95% CI]	21.2 [19.3–23.1]	11.1 [9.7–12.7]	
HbA1c < 7%, % [95% CI]	57 [54.7–59.3]	57 [54.6–59.4]	0.999
Hypertension, % [95% CI]	80.4 [78.3–82.4]	67.7 [65–70.3]	< 0.001
SBP, mmHg, mean (SD)	133.9 (11.9)	132.5 (11.6)	0.002
DBP, mmHg, mean (SD)	76.5 (6.7)	76.8 (7.1)	0.214
Hypercholesterolemia, % [95% CI]	42.8 [40.4–45.3]	31.5 [29.1–34]	< 0.001
LDL-Cholesterol, mg/dl, mean (SD)	113.53 (26.4)	110.4 (25.1)	0.001
Triglycerides, mg/dl, mean (SD)	140.5 (71.5)	141.4 (80.7)	0.739
GFR, ml/min/1.73 m2, mean (SD)	70.5 (17.1)	77.5 (15.9)	< 0.001
Albuminuria, mg/dl, mean (SD)	23.8 (76.6)	34.3 (98.7)	0.013
Heart failure, % [95% CI]	5.7 [4.5–7]	3.5 [2.6–4.8]	0.014
Atrial Fibrillation, % [95% CI]	16.5 [14.9–18.3]	14.0 [12.5–15.8]	0.04
Diabetic neuropathy, % [95% CI]	9.3 [8.0–10.7]	7.1 [5.9–8.4]	0.02
Diabetic retinopathy, % [95% CI]	7.6 [6.5–8.9]	9.3 [8.0–10.8]	0.08
Peripheral arteriopathy, % [95% CI]	3.4 [2.6–4.3]	12.3 [10.8–13.9]	< 0.001

SD: standard deviation; CI, confidence interval; IQR, interquartile range; BMI, body mass index; SBP, systolic blood pressure; DBP, diastolic blood pressure; LDL-cholesterol, low-density lipoprotein cholesterol; GFR, glomerular filtration rate

Compared with noncases, cases of incident stroke or TIA differed by sex in terms of the following variables: age and hypertension in both sexes and BMI in men (Table 2).

**Table 2:** Baseline characteristics by incidence of first stroke or transient ischaemic attack (TIA) during follow-up

	Females			Males		
	Stroke/TIA	No stroke/TIA	*p* value	Stroke/TIA	No stroke/TIA	*p* value
	(n = 156)	(n = 1598)		(n = 147)	(n = 1541)	
Age (years), mean (SD) [95% CI]	73.7 (8.8)	68.5 (10.8)	< 0.001	70.3 (9.4)	64.8 (11.1)	< 0.001
Diabetes duration (years), median [IQR]	6.7 (4.7)	6.4 (4.7)	0.939	6.6 (5.4)	6.0 (4.6)	0.093
BMI (kg/m^2^), mean (SD)	30.9 (5.9)	31.1 (5.4)	0.695	29.1 (4.0)	29.8 (4.4)	0.038
< 25 kg/m^2^, %	13.5	11.2	0.713	17.0	11.9	0.033
25–30 kg/m^2^, %	35.9	33.9		49.0	45.3	
31–35 kg/m^2^, %	30.1	33.7		24.5	31.5	
> 35 kg/m^2^, %	20.5	21.2		9.5	11.3	
HbA1c < 7%, %	51.4	57.5	0.209	47.2	57.6	0.084
Hypertension, %	88	79	0.048	80.3	67	0.024
Hypercholesterolemia, %	45.1	42.6	0.606	30.6	31.6	0.853
Heart failure, %	8	5.5	0.290	5.5	3.4	0.426
Atrial Fibrillation, %	22.2	16.1	0.104	11.7	13.8	0.279
Diabetic neuropathy, %	11.5	9.1	0.312	7.5	7.1	0.853
Diabetic retinopathy, %	11.5	7.3	0.055	12.9	9.0	0.198
Peripheral arteriopathy, %	5.1	3.2	0.200	15.6	11.9	0.191

SD: standard deviation; CI, confidence interval; IQR, interquartile range; BMI, body mass index

Table 3 shows the characteristics of the variables analysed in the longitudinal submodels differentiated by sex. Women had more measurements for all variables except albuminuria, with blood pressure having the highest number of measurements for both sexes.

**Table 3:** Characteristics of the variables analysed in the longitudinal submodels by sex: record counts and descriptive statistics

	Females									Males									*p value*
	(n = 1754)									(n = 1688)									
	n*	n/p**	min	P5	P25	Median	P75	P99	max	n*	n/p**	min	P5	P25	Median	P75	P99	max	
Anthropometric variables																			
SBP (mmHg)	35,677	14	80	110	120	130	140	172	230	31,872	14	78	110	120	130	140	170	240	< 0.001
DBP (mmHg)	35,677	14	40	60	70	71	80	86	120	31,872	14	35	60	70	72	80	98	140	0.597
Laboratory variables																			
HbA1c (%)	21,060	11	4.1	5.7	6.4	6.9	7.7	11.2	17.2	19,660	11	4.1	5.6	6.3	6.9	7.6	11.2	15.6	< 0.001
GFR (ml/min/1.73 m^2^)	22,429	12	3.2	34.9	57.4	73.1	85.8	107	124.5	20,267	12	4.4	39.3	63.4	79	89.9	109	126	< 0.001
LDL-Cholesterol (mg/dl)	19,846	11	20.5	57	81	99	120	189	273	18,081	10	10.4	55	77	94.6	115	176	266	< 0.001
Triglycerides (mg/dl)	22,099	12	25	65	94	125	170	401	968	20,165	12	30	58	86	117	166	484	999	< 0.001
Albuminuria (mg/dl)	8,077	4	2.4	3.2	5	6.1	16	363	979	8,332	4	2.5	3.4	5	9.6	27.2	500	960	< 0.001

SBP, systolic blood pressure; DBP, diastolic blood pressure. GFR, glomerular filtration rate; LDL cholesterol, low-density lipoprotein cholesterol; Min, minimum value; Max, maximum value

P5: 5th percentile. P25: 25th percentile. P75: 75th percentile. P99: 99th percentile

^*^ n = total number of records during follow-up; ** n/p = median number of records per subject during monitoring

During the 12 years of follow-up, 303 (8.8%) cases of first stroke or TIA, 128 (3.7%) cases of first myocardial infarction, and 551 deaths (16.0%; 272 among men (16.1%), and 279 among women (15.9%)) were reported.

Table 4 presents the predictive models for both sexes. In males, age was identified as an independent predictor, whereas it exhibited a positive interaction with AF in females. The final model for males included AF, SBP and DBP, with albuminuria and the GFR as adjustment variables. For females, the model incorporated AF, renal function (measured by albuminuria and the GFR), and blood pressure, whereas HbA1c and LDL-C levels were used as adjustment variables. Both models demonstrated an AUC greater than 0.70.

**Table 4:** Predictive variables of a first stroke or TIA according to sex in the MADIABETES cohort

Male					Female				
	HR	CI 95%		*p* value		HR	CI 95%		*p* value
Age > = 76 years	1.87	1.09	3.12	0.023	Age > = 81 years	1.11	0.55	2.12	0.746
Atrial fibrillation (yes/no)	1.81	1.10	2.87	0.020	Atrial fibrillation (yes/no)	1.58	0.94	2.56	0.081
Area (1/Albuminuria)^a^	1.02	0.99	1.05	0.199	Interaction age (> = 81 years) and atrial fibrillation	4.15	1.51	11.44	0.006
Area (GFR^b^), t* = 7	1.01	0.99	1.02	0.197	Area (1/Albuminuria)^a^, *t* = 3	1.05	1.08	1.01	0.010
Area (log (SBP)^c^, t* = 3	1.04	1.01	1.07	0.006	Area (GFR)^b^, t* = 5	1.02	1.03	1.01	< 0.001
Area (sqrt (DBP)^d^, t* = 7	0.59	0.33	1.05	0.068	Area (log (SBP)^c^, t* = 5	1.03	1.00	1.07	0.042
					Area (sqrt (DBP)^d^, t* = 3	0.40	0.20	0.81	0.010
					Area (1/HbA1c)^e^	1.07	0.99	1.19	0.148
					Slope (sqrt(LDL-C)^f^, t* = 7	2.31	0.91	5.95	0.082

HR: Hazard ratio; CI: Confidence interval; GMR, glomerular filtration rate; SBP, systolic blood pressure; DBP, diastolic blood pressure

*Calculation performed with the values of the last *t*-years of the follow-up period that improves the discriminative capacity of the model

^a^for a 0.01-unit decrement in the area under the profile of albuminuria (mg/dL)^−1^

^b^for a 1-unit decrement in the area under the profile of glomerular filtrate rate (mL/min/1,73 m^2^)

^c^for a 0.01-unit increment in the area under the profile of log(SBP mmHg)

^d^for a 1-unit increment in the area under the profile of sqrt(log(BSPmmHg))

^e^for a 0.01-unit decrement in the area under the profile of HbA1c(%)^−1^

6^f^for a 1-unit increment in the slope the profile of sqrt(LDL-C mg/dl)

Although the follow-up period was 12 years, the main variables (SBP and DBP, albuminuria and GFR, and LDL-C levels) showed greater predictive ability when values from the last 3 to 7 years were considered.

A practical example of the dynamic prediction of the risk of developing a first stroke or TIA in a 72-year-old man with T2DM over a 7-year follow-up period is presented in Supplementary Fig. 2.

The internal validation results for the predictive performance metrics are presented in Supplementary Fig. 3. The area under the receiver operating characteristic curve (AUCt,u) was slightly greater in males than in females. However, the Brier score, which indicates prediction error (PEt,u), was higher in females than in males. The calibration curve was similar for both sexes.

## Discussion

Here, we reported that the crude incidence rates of stroke and TIA per 100,000 person-years were similar to those reported in other studies [8, 28, 29]. However, in our study, women had a lower crude incidence rate than men did, as opposed to a previous meta-analysis of 64 cohorts [30]**.** However, the Atherosclerosis Risk in Communities Study showed an adjusted risk ratio for stroke of 1.33 among men compared with women [31].

HbA1c is a well-established, independent predictor of future strokes in patients with T2DM [8]. Two meta-analyses of stroke risk factors revealed that each 1% increase in HbA1c was associated with a significant increase in stroke incidence—specifically, an 11% increase [32]] and a 17% increase [33]—after controlling for potential confounding factors.

Several biological mechanisms may explain the potential direct link between chronically elevated blood glucose levels and vascular disease. Persistently high blood glucose levels promote endothelial dysfunction [34], increased arterial thickness, plaque formation, and atherosclerosis [35]. Additionally, diabetic macroangiopathy and microangiopathy exacerbate each other. Macrovessel obstruction may lead to brain perfusion deficiency and microvascular diseases. Microvascular dysfunction also affects macrovessel collateral circulation, increases the risk of stroke, and worsens prognosis [36]. The physiological processes that are thought to contribute to vascular disease do not occur suddenly but develop gradually and cumulatively over decades of exposure to persistently high blood glucose levels. This is the reason why an induction period is necessary. Additionally, insulin resistance is common in T2DM patients and serves as an additional risk factor for ischemic stroke [37]. It is more prevalent in women, particularly those with a sedentary lifestyle, during menopause, or with polycystic ovarian syndrome.

However, our data indicate a nonsignificant association between HbA1c levels and stroke incidence. Among female patients, the association reached borderline significance (HR = 1.07; 95% CI 0.99–1.19), which is consistent with the findings of previous studies [38]. Several factors may explain why glucose levels have a stronger adverse effect in women than in men. One reason is that the risk of ischaemic stroke increases with lower fasting glucose levels in women than in men, even after adjustment for treatment with oral medication or insulin [39]]. Additionally, women with diabetes often have significantly higher blood pressure and lipid levels than their male counterparts do [40]. Moreover, it has also been suggested that treatment bias may contribute to the increased risk associated with diabetes in women, with men being favoured in terms of care. Recent research has shown that women with diabetes or vascular disease are less likely than men to receive treatments such as aspirin, statins, or antihypertensive drugs [41].

Global metabolic control is more important than glycemic control alone in stroke development. This situation may be because antidiabetic drugs, such as SGLT2 inhibitors (in people under 75) and GLP-1 receptor agonists, can reduce the risk of stroke through several mechanisms. These mechanisms include reducing inflammation, reducing insulin resistance (often higher in women, as indicated by the triglyceride‒glucose index), minimizing cell death, and promoting autophagy [41–44]**.**

The significant impact of AF on the risk of stroke has been recognized for at least 30 years [45]. While AF typically increases the risk of stroke by up to five times in the general population, the presence of diabetes mellitus increases this risk by an additional 2–3.5% [46]. This increase is attributed to chronic hyperglycemia, which plays a critical role in atrial remodelling and the deelopment of AF [47]. This study confirms the importance of this risk factor and its interaction with age in women. This study also highlights the need for appropriate management of AF and reversal or anticoagulation therapy in patients of advanced age at the end of life. This increased risk is for ischaemic stroke, but in our study, it was not possible to differentiate by type of stroke. However, there is significant evidence indicating that ischaemic strokes are more prevalent than haemorrhagic strokes in patients with diabetes mellitus. In fact, the ratio of ischaemic to haemorrhagic strokes is notably greater in patients with diabetes than in the general population [48]. Additionally, lacunar stroke (small vessel disease) is the most common type of stroke observed in patients with diabetes [49].

A recent observational study by Rawshani et al. [8] in a population with T2DM reported an association between LDL cholesterol levels and the risk of stroke. However, that study revealed that LDL cholesterol was less significant in predicting stroke than other factors, such as SBP, AF, smoking, glycated haemoglobin and duration of diabetes. Typically, LDL cholesterol levels are more strongly associated with coronary events than with stroke. This situation is partly because fewer than half of all strokes are caused by large vessel atheromas, whereas the majority are caused by nonatheromatous factors such as arrhythmias and small vessel disease. In this sense, LDL control has only shown a significant benefit in preventing stroke recurrence in atherothrombotic stroke patients [50]. Additionally, a recent meta-analysis has shown that intensive lipid-lowering treatment to reduce LDL cholesterol is linked to a decreased risk of recurrent stroke in trials involving participants with evidence of atherosclerosis. The risk ratio (RR) for this group was 0.79, with a 95% CI of 0.69–0.91. However, this association was not observed in trials where most participants did not have evidence of atherosclerosis, which had an RR of 0.95 and a 95% CI of 0.85–1.07 [51].

We found a nonsignificant trend toward an association between LDL cholesterol and stroke in women but not in men. This may be related to the different patterns of stroke observed between the sexes, as AF and hypertension are more common among female patients with stroke [52]. In addition, a greater proportion of strokes in women than in men are attributed to cardioembolic causes [53].

Traditionally, both SBP and DBP have been regarded as independent predictors of vascular events and mortality [53–55]. However, our study suggests that an increase in DBP may serve as a protective factor against the incidence of first strokes in patients at high vascular risk, such as those with T2DM. Several explanations can be offered for this finding. First, in our cohort, DBP values fell within a narrow range, primarily on the lower side, with medians of 71 mmHg for women and 72 mmHg for men. Only 25% of the participants had a diastolic pressure above 80 mmHg. This pattern contrasts with findings from other cohorts, as the MADIABETES cohort consists of well-controlled patients. Second, some observational studies have indicated a protective benefit against ischemic stroke for those with a DBP > 70 mmHg; however, this protective effect diminishes at levels of 90 mmHg and above. The Franklyn study examined 791 participants who had survived their first cardiovascular disease (CVD) event and were followed for five years. The study revealed that those with DBP values below 70 mmHg had a significantly greater risk of experiencing a stroke, with an HR of 3.7 (95% CI 2.4–5.7), than those with DBP values between 70 and 89 mmHg, according to an adjusted Cox regression model [56].

The HOT trial [57] revealed that participants with a target DBP ≤ 85 mmHg had 4.7 strokes per 1000 patient-years, whereas those with a target DBP ≤ 90 mmHg had 4.0 strokes per 1000 patient-years. However, there are few recommendations on the optimal target DBP to reduce the risk of first stroke in patients with diabetes or high vascular risk. For example, the 2024 Guidelines for the Primary Prevention of Stroke [58] recommend a target SBP of 130–134 mm Hg versus 140–144 mm Hg on the basis of a recent network meta-analysis, but no recommendation was made on the optimal target BDP. It is possible that we are missing the right part of the J-curve described by other investigators [59] in our population with tight control of blood pressure values, thus resulting in a reduced stroke risk for the upper values of DBP. However, lower values of DBP might also be indicative of an abnormally stiff and already diseased vasculature or markers of underlying comorbid diseases that cause both low DBP and increased vascular morbidity [60].

An initial standard analysis of the MADIABETES cohort did not reveal an effect of blood pressure on the development of stroke or TIA [9]. A plausible explanation for this finding could be motived by the differences in the statistical approaches used. The time-varying Cox model uses the last-observation-carried-forward method, which assumes that the values of time-varying covariates remain constant between measurements. This assumption is unrealistic for risk factors such as SBP and DBP, which change dynamically over time and can be influenced by previous events. This can lead to misleading results. In contrast, a joint model analysis more accurately captures the biological processes at work, as it considers the evolution of a patient's condition over time. Furthermore, valid conclusions can only be drawn from a joint distribution of longitudinal measures and the missingness process when dealing with unavoidable missing data. In the context of endogeneity and informative missingness, the joint modelling framework provides a solution by proposing a relative risk model for the time-to-event outcome that is based on the true underlying value of the longitudinal measurement [61]. These advantages make joint models versatile and powerful tools for analysing complex medical data. Their ability to adapt to changing patient conditions and covariates over time makes them particularly well suited to scenarios where such changes are frequent and significant.

As previously highlighted, our results underscore the critical importance of values obtained in the last 3–7 years, after 12 years of follow-up, in predicting the event. This underlines the urgency of better control of parameters such as HbA1c, blood pressure and LDL cholesterol, even in the later stages of a disease such as diabetes.

Patients suffering from kidney disease, irrespective of etiology, face a significantly elevated risk of myocardial infarction and are up to fourfold more prone to experiencing a stroke compared to those without renal disorders. Women are especially vulnerable to this risk compared to men, and this risk escalates as kidney function deteriorates. Diabetic nephropathy is the most common underlying cause of kidney disease [62]. The overall impact of diabetes mellitus on the development of stroke is presumably due to microvascular damage and the resulting renal failure. A study of patients with atherothrombotic disease found that chronic kidney disease (CKD) was associated with an increased risk of ischemic stroke or transient ischemic attack (TIA), with a hazard ratio (HR) of 1.54 (95% confidence interval 1.13–2.09) for those with CKD compared to those without [63]. This association is further reinforced by the prevalence of both diabetes and atherothrombotic disease, suggesting a shared pathophysiological mechanism. The potential contributors to this increased risk include anemia, oxidative stress, impaired calcium-phosphate homeostasis, and inflammation, which can adversely affect renal function and contribute to an elevated stroke risk.

The role of renal function as a predictor of various stroke subtypes in patients with type 2 diabetes remains to be less studied in comparison to the extensive research conducted on type 1 diabetes. Diabetic nephropathy has been shown to elevate the risk of both ischemic (lacunar and non-lacunar) and hemorrhagic strokes, with incidence rates comparable across individuals with type 1 and type 2 diabetes, as well as the general population. The probability of experiencing either type of stroke increases from the early stages of proteinuria to advanced kidney disease [64]**.** Notably, the proportion of lacunar infarcts among patients with diabetes is higher than that observed in the general population, although a direct link between this and the severity of diabetic nephropathy remains to be established. Asymptomatic cerebral small vessel disease, diagnosed through magnetic resonance imaging, has been associated with CKD, irrespective of the presence of diabetes.

Elderly stroke patients with kidney dysfunction face significantly higher risks of both short- and long-term mortality. In contrast, younger patients (ages 15–49) who have experienced an ischemic stroke demonstrate that both low and high glomerular filtration rates (GFR) are independent predictors of long-term mortality [65]. This relationship may be influenced by age; for instance, patients with type 1 diabetes and hypertension typically present with low GFR due to advanced kidney disease. Conversely, patients with type 2 diabetes are often associated with high GFR, indicating they may be at an earlier stage of diabetic vascular damage that reflects renal hyperfiltration prior to the onset of progressive kidney disease.

To apply our findings to the general population, we need to validate them externally in a new cohort that continuously records the risk factors included in our model. Once this validation is complete*,* we can implement our model into routine clinical practice, as the necessary statistical packages and software, such as R and the JMbayes2 (v4.1) package, are freely available [27].

### Strengths and limitations

Potential limitations of this study must be considered. First, our data did not consider the various subtypes of strokes, which can have different underlying mechanisms and risk factors. This limitation arose because the study was conducted in a primary care setting, where access to imaging studies or detailed reports from neurologists was not always feasible. Second, our cohort consists of a well-controlled sample of patients with T2DM, which may limit the generalizability of our findings to the broader population with poorly controlled HbA1c, blood pressure, and LDL-cholesterol levels. Third, the narrow ranges for LDL cholesterol (IQR, 39 mg/dl in men and 38 mg/dl in women) and HbA1c (IQR, 1.3% in both sexes) made it difficult to achieve statistical significance in risk prediction with the sample size used.

However, the prospective design and methodology of this study, which considers a joint model for longitudinal and time-to-event data, strengthens our findings. In addition, the gender perspective was used to study stroke-associated factors by sex, as recommended in international guidelines (53), and the data obtained from current medical practice in our setting provide us with an overall view of the actual outcomes of patients with T2DM in our daily clinical practice.

## Conclusions

We have introduced a new perspective to the study of stroke predictors by using joint models for longitudinal and time-to-event data within a cohort of people with T2DM. This approach effectively captures the underlying biological processes by considering how a patient's condition evolves over time.

Our findings align with those of previous epidemiological studies, highlighting the significance of AF as a predictive factor and its interaction with age in women. Additionally, the results underscore the importance of specific risk factors, such as SBP in both sexes and the glomerular filtration rate in men. Interestingly, an increase in DBP appeared to act as a protective factor. This observation may be attributed to the narrow range of DBP among the patients studied, suggesting that further research could explore this finding in greater depth. These factors were particularly relevant during the last 3–7 years of the 12-year follow-up period.

## Supplementary Information

Supplementary file 1.

Supplementary file 2.

Supplementary file 3.

## Data Availability

The database is not available to the public, and its use is restricted to research groups affiliated with the MADIABETES consortium, IdIPAZ Health Research Institute, La Paz University Hospital, and IMDEA Food Institute, Madrid (Spain).

## Abbreviations

AF: Atrial fibrillation
AUC: Area under the curve
BMI: Body mass index
CI: Confidence interval
CKD: Chronic kidney disease
CVD: Cardiovascular disease (coronary heart disease, stroke, or peripheral arterial disease), otherwise known as atherosclerotic vascular disease (AVD)
CT: Computerized tomography
DBP: Diastolic blood pressure
DIC: Deviance information criterion
GFR: Glomerular filtration rate
HbA1c: Hemoglobin A1c
HR: Hazard ratio
INE: National institute of statistics
IDF: International diabetes federation
IQR: Interquartile range
LDL-C: Low-density lipoprotein cholesterol
MRI: Magnetic resonance imaging
ROC: Receiver operating characteristic
SBP: Systolic blood pressure
TIA: Transient ischaemic attack
T2DM: Type 2 diabetes mellitus
