# Supplementary material for: A different perspective on studying stroke predictors: joint models for longitudinal and time-to-event data in a type 2 diabetes mellitus cohort
Source: Cardiovasc Diabetol. 2025 Apr 16;24:165. doi: 10.1186/s12933-025-02713-9 (PMC12004838; doi:10.1186/s12933-025-02713-9)
Supplement: Supplementary file 1 [file 12933_2025_2713_MOESM1_ESM.docx]

Albuminuria

Males

Females

**Supplementary Figure 1. Boxplots of longitudinal variables by sex and event (stroke/TIA)***

Glomerular Filtration Rate

HbA1c

LDL-chol

Triglycerides

SBP

DBP

* TIA: Transient ischemic attack. Females: green boxes; Males: blue boxes. Patients with stroke/TIA: red boxplot. Patients without stroke/TIA: gray boxplot.

Original values (white background): albuminuria (mg/dl); GFR (glomerular filtration rate, ml/min/1.73 m^2^); HbA1C (%); LDL-Cholesterol (mg(dl); Triglycerides (mg/dl); SBP (systolic blood pressure, mmHg); DBP (diastolic blood pressure, mmHg).

Transformed values (yellow background): inverse of albuminuria (1/albuminuria); inverse of HbA1C (1/HbA1C); square root of LDL-Cholesterol; inverse of square root of LDL-Cholesterol; logarithm of SBP (logSBP) and square root of DBP, respectively.

Translated with www.DeepL.com/Translator (free version)
