# Supplementary material for: A different perspective on studying stroke predictors: joint models for longitudinal and time-to-event data in a type 2 diabetes mellitus cohort
Source: Cardiovasc Diabetol. 2025 Apr 16;24:165. doi: 10.1186/s12933-025-02713-9 (PMC12004838; doi:10.1186/s12933-025-02713-9)
Supplement: Supplementary file 3 [file 12933_2025_2713_MOESM3_ESM.docx]

**Supplementary Figure 3. Internal validation results for predictive performance metrics.**

**Calibration plot**

**Brier score (PE_t,u_)**

**AUC_t,u_**

Area under the receiver operating curve (AUC_t,u_), Brier score (prediction error, PE_t,u_) and calibration plot. The boxplots includes the overfitting-corrected estimates of these measures from each bootstrap iteration (n=100) performed on a sample of 1000 patients drawn from the cohort of male and female, cohort respectively, on which the original models were obtained. We assumed all the repeated measurements up to the follow-up time t= 7 years and the future time point u=10 years.
